# First report of the occurrence of Asian citrus psyllid *Diaphorina citri* (Hemiptera: Liviidae), an invasive species in Nigeria, West Africa

**DOI:** 10.1038/s41598-020-66380-4

**Published:** 2020-06-10

**Authors:** Abiola Olufunke Oke, Abiola Adeyinka Oladigbolu, Madhurababu Kunta, Olufemi J. Alabi, Mamoudou Sétamou

**Affiliations:** 1 National Horticultural Research Institute (NIHORT), Idi-Ishin Jericho Reservation Area, P.M.B, 5432 Ibadan, Nigeria; 2 Texas A&M University-Kingsville Citrus Center, Weslaco, 78599 US; 3 Department of Plant Pathology & Microbiology, Texas A&M AgriLife Research & Extension Center, Weslaco, TX 78596 US

**Keywords:** Entomology, Invasive species

## Abstract

The Asian citrus psyllid (ACP; *Diaphorina citri*) is the vector of *Candidatus* Liberibacter asiaticus (CLas) that is associated with the devastating Huanglongbing (HLB; citrus greening disease). This pest of Asian origin has spread into the Americas and more recently into a few countries in East Africa. During recent surveys, suspect ACP adults and nymphs were recorded for the first time infesting citrus trees in southwest Nigeria. Morphological identification and DNA barcoding confirmed the samples to be *D. citri*. Analysis of the obtained sequences revealed that the ACP recorded in Nigeria clustered with other taxa in the previously identified B1 clade that consists of populations from different continents. The presence of the endosymbionts *Ca*. Carsonella ruddii and *Ca*. Profftella armatura in ACP from Nigeria was also confirmed by PCR and Sanger sequencing. The ACP individuals were assayed for the presence of CLaf, CLam and CLas by qPCR, but none of the insects tested positive for any of the Liberibacters. The prolific nature of ACP and the tropical climate prevailing in the citrus-producing areas of Nigeria and other West African countries may favor its rapid spread and population increase, thus posing a grave threat to the sustainability of citriculture in these countries.

## Introduction

Citrus is one of the world’s most important economic crops and the most widely grown fruit tree in Africa. Nigeria is the largest citrus producer in Africa and the ninth in the world with a production of about 4,088,994 tons from approximately 839,628 ha of groves in 2017 (FAO 2019). Oranges account for most of the citrus grown in the country, but significant quantities of grapefruit, tangerine, lime and lemon are also cultivated^1^. During the past four decades, citrus acreage and production have dramatically increased in Nigeria due to the suitable ecological and climatic conditions for its production and the establishment of the National Horticultural Research Institute (NIHORT) in 1975 to promote the horticultural sector of the country, with citrus as a mandate crop^2^.

This significant growth in citrus production in Nigeria stemmed mainly from an increase in acreage, as yields have remained mostly stagnant, not exceeding 4–5 tons/ha and far below the world average of 14–15 tons/ha^3^. Among the limiting factors of citrus productivity in Nigeria is the absence of large commercial production. Most of the citrus is grown by smallholder famers in mixed cropping systems with cocoa, kola, coffee or rubber, and with minimal or no investment in agrochemicals and other inputs^1^. Given the favorable tropical climate in the citrus production areas of Nigeria, arthropod pests and diseases may also constitute a major impediment to enhanced productivity. Among the arthropod pests affecting citrus, the sap sucking hemipteran pests are of great concern. Feeding damage by these insects due to uptake of plant sap causes distortion, wilting, premature leaf drop, reduction of fruit sizes, and occasional tree death^4^. Furthermore, some of these sap-sucking pests are documented vectors of pathogens of economically important citrus diseases such as Tristeza, Citrus Variegated Chlorosis, and Huanglongbing (HLB) or Citrus Greening, among others^5,6^.

Although citrus tristeza virus (CTV) has killed more than 50 million citrus trees on sour orange rootstock since the beginning of the epidemics in the 1930s in Argentina and Brazil^5^, HLB is undoubtedly now the most economically important citrus disease in the world^6,7^. HLB is a highly destructive and fast spreading disease that is affecting citrus industries in Africa, the Americas and Asia^6–8^. Three related phloem-inhabiting fastidious bacteria: *Candidatus* Liberibacter africanus (CLaf), *Ca*. Liberibacter americanus (CLam) and *Ca*. Liberibacter asiaticus (CLas) are consistently detected in HLB-affected citrus trees^6^. These bacteria are transmitted via clonal propagation or grafting of infected material and by two psyllid vectors. The African triozid (*Trioza erytreae* (Del Guercio) (Hemiptera: Triozidae)) is the primary vector of CLaf but it can also transmit CLas, while the Asian citrus psyllid (*Diaphorina citri* Kuwayama (Hemiptera: Liviidae)) is the vector for CLas and CLam^9^. To date, CLaf and CLam are restricted to the African and American continents, respectively, while CLas is widespread across countries in Asia, the Americas, and more recently in Africa^10,11^.

*D. citri* has invaded the Americas in the last two decades^10,12–16^. The rapid spread of CLas and its ensuing HLB is due primarily to the prolific and invasive nature of *D. citri*^17^. *Diaphorina citri* has also been reported more recently in Tanzania^18,19^ in East Africa, indicating its global spread. The presence of *D. citri* in East Africa poses significant risks of further spread across the African continent due to the increase in international and transcontinental trade. In addition to its invasive potentials, ACP can hitchhike budwood, rootstock seedling or nursery plants of rutaceous hosts^19^ transported across national and international borders. Post-introduction, the favorable climatic conditions prevailing across citrus-producing regions of Africa could favor its establishment and population growth.

Knowledge of the presence of *D. citri* as early as possible would facilitate the development and implementation of targeted pest and disease mitigation efforts to limit its spread and establishment. Despite being a major citrus-producing country in Africa, no previous studies have been conducted to determine the presence of *D. citri* in Nigeria. To fill this void, citrus commodity pest surveys were conducted in southwestern Nigeria to determine the presence or absence of *D. citri* and if present, to diagnose the sampled individuals for Liberibacter.

## Results

### Field detection and morphological identification

**Figure 1:**
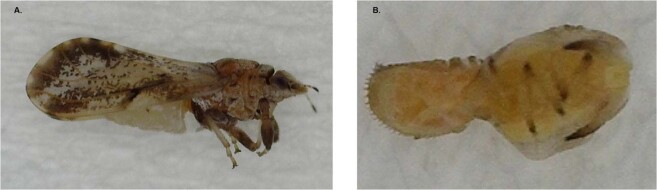
Adult (**A**) and nymph (**B**) of the Asian citrus psyllid (*Diaphorina citri* Kuwayama) detected in different locations (Table 1) in Oyo state, Nigeria.

The 10 surveyed sites (groves = 4, backyards = 6) were all within altitudes ranging from 152 to 275 m above sea level. Using previously described features^20–22^, and based on comparisons with voucher specimens, the field-collected insect samples were morphologically identified as *D. citri* (Fig. 1). Voucher specimens of these samples have been deposited at the TAMUK-CC in Weslaco, Texas Entomology Laboratory, and at NIHORT, Ibadan, Nigeria. During the survey, *D. citri* adults were observed feeding on mature and/or young expanding citrus leaves from 80% of the sites (8 out of 10; grove = 2, backyard = 6), while nymphs were recovered only from one backyard site (10% of all sites). *D. citri* was not observed in two of the four surveyed groves. A total of 248 individuals (adult = 216, nymph = 32) were collected from the eight infested sites (Table 1). During the survey none of the inspected trees showed symptoms of infestation of African triozid, *Trioza erytreae* (Del Guercio) (Hemiptera: Triozidae) such as open galls on leaves associated with its feeding damage. In addition, no individual of the African triozid, was encountered. Considering the wide spatial distribution of the positive detection sites, and the observation of developing nymphs at one site, it is likely that *D. citri* is established and relatively widespread in Oyo state.

**Table 1:** Field attributes of suspect Asian citrus psyllid (*Diaphorina citri* Kuwayama) individuals sampled from different locations across Oyo State, Nigeria.

Locationa	Host plants	Coordinate	Altitude (m)	Citrus system	Adults	Nymphs
NIHORT, Ibadan	Sweet orange	N07°24ʹ19.9″ E003°50ʹ58.6″	196	Backyard	24	0
NIHORT, Ibadan	Sweet orange	N07°24ʹ12.0″ E003°50ʹ58.2″	188	Grove	0	0
NIHORT, Ibadan	Grapefruit and lemon	N07°24ʹ22.2″ E003°50ʹ48.6″	168	Grove	0	0
Agbofieti, Ibadan	Sour Orange	N07°24ʹ55.7″ E003°49ʹ05.4″	192	Backyard	35	0
Idi-Ishin, Ibadan	Grapefruit	N07°24ʹ06.9″ E003°51ʹ32.9″	172	Backyard	54	32
Oyo town	Grapefruit and Sweet orange	N07°48ʹ50.3″ E003°54ʹ43.4″	275	Backyard	13	0
Adejumo (Igana)	Grapefruit and Sweet orange	N07°52ʹ05.4″ E003°13ʹ07.7″	182	Grove	41	0
Tudi	Grapefruit and Sweet orange	N07°51ʹ14.8″ E003°10ʹ39.7″	195	Grove	27	0
Eruwa	Sweet orange	N07°32ʹ47.6″ E003°26ʹ43.3″	152	Backyard	5	0

^a^NIHORT, National Horticultural Research Institute, Ibadan, Nigeria.

### Molecular detection

Gene-specific DNA amplicons of the expected sizes (Table 2) were obtained from a subset of randomly selected 13 *D. citri* (9 adults and 4 nymphs) that were representative of the spatial diversity of the sampled insects. A total of 13 COI-specific (GenBank accession nos. MT040168 - MT040180), 4 argH-specific (MT036086 - MT036089), and 6 atpA-specific (MT040183 - MT040188) sequences were obtained. The BLASTN analysis of these sequences produced significant (≥99% nt identity; 100% query coverage; E-value 0.0) matches to corresponding gene-specific sequences of *D. citri*, *Ca*. Profftella armatura, and *Ca*. Carsonella ruddii available in GenBank from different countries. In pairwise comparisons, the *D. citri*-specific COI sequences derived in this study shared 99–100% nt identities among themselves and the same range of nt identities with corresponding global sequences of this psyllid species; the COI-specific *D. citri* sequences were significantly distinct (42–43% nt identity) from corresponding sequences of GenBank isolates of *T. erytreae*. The *Ca*. Carsonella ruddii *argH*-specific sequences derived in this study shared 99.8–100% nt identity with each other and 97–100% nt identity with corresponding global sequences of this psyllid P-endosymbiont. The *Ca*. Profftella armatura *atpA*-specific sequences derived in this study shared 99.5–100% nt identity with each other, and 99.5–99.8% nt identity with corresponding global sequences of this psyllid S-endosymbiont. These results provide definitive confirmation of the identity of the insect individuals as *D. citri*. They also showed that *D. citri* individuals from Nigeria carry the same primary and secondary bacterial endosymbionts species in their bacteriome as previously documented from other citrus-producing areas of the world^9,23,24^.

**Table 2:** Primers used for the amplification of gene segments of the Asian citrus psyllid (*Diaphorina citri*) and its endosymbionts *Candidatus* Carsonella ruddii and *Ca*. Profftella armatura.

Primer name	Sequence (5′ – 3′)	(bp)	Host/Gene Target	Source
DCITIR COI-L	AGGAGGTGGAGACCCAATCT	834	*D. citri*/mtCOI	Boykin *et al*. 2012
DCITRI COI-R	TCAATTGGGGGAGAGTTTTG			
argH-F1	CTCCTATGCCTGGATTTACTCA	887	*Ca*. Carsonella ruddii/*argH*	Wang *et al*. 2017
argH-R1	TTGATTAGGCGCTGTACCTCC			
atpA-F1	CAATAATCGGTATCGCTGTT	753	*Ca*. Profftella armatura/*atpA*	Wang *et al*. 2017
atpA-R1	AGCATATTACGGAAGGTGAT			

### Phylogenetic analysis

**Figure 2:**
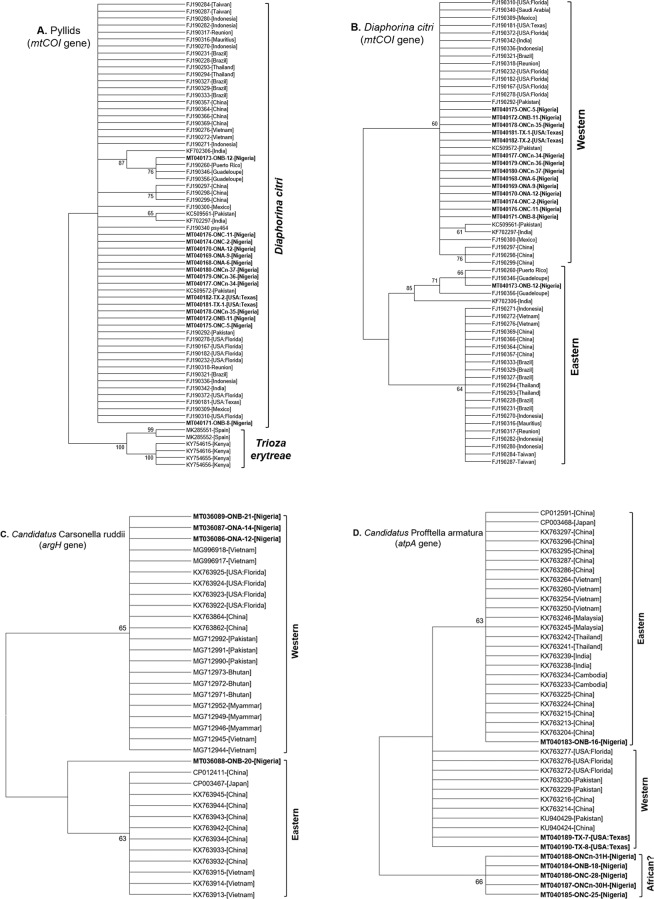
Maximum Likelihood (ML) phylogenetic trees depicting the evolutionary relationships between adults and nymphs of the Asian citrus psyllid (*Diaphorina citri* Kuwayama), and their primary and secondary endosymbionts, sampled from different locations (Table 1) in Oyo state, Nigeria and corresponding sequences of global populations of each organism. The ML trees were derived based on analyses of sequences specific to the *mtCOI* gene of *D. citri* (**A** and **B**); MT040168 - MT040182 derived in this study; others from GenBank), the *argH* gene of the primary endosymbiont *Ca*. Carsonella ruddii (**C**); MT036086 - MT036089 derived in this study; others from GenBank), and the *atpA* gene of the secondary endosymbiont *Ca*. Profftella armatura (**D**); MT040183 - MT040190 derived in this study; others from GenBank). The Tamura 3-parameter was determined as the model with the lowest BIC (Bayesian Information Criterion) scores and was therefore used in ML phylogenetic analysis for each of the gene-specific sequences (with 1,000 bootstrap replications). Branches with <60% bootstrap support were collapsed.

The Tamura 3-parameter was determined as the model with the lowest BIC (Bayesian Information Criterion) scores and was therefore used in maximum likelihood (ML) phylogenetic analysis of each of the gene-specific sequences. As expected, the *mtCOI* sequences from Nigeria clustered within the *D. citri* clade, distinct from the *T. erytreae* clade on the psyllid ML tree (Fig. 2A). Further analyses revealed a clear segregation of the *D. citri*-specific *mtCOI* sequences into the previously defined Western and Eastern clades^25^ with strong (>60%) bootstrap support (Fig. 2B). Most of the adult and nymph *mtCOI* sequences from Nigeria (MT040168 - MT040172 and MT040174 - MT040180) (n = 12) segregated into the Western clade; only one sequence (MT040173 from Agbofieti, Ibadan) clustered into the Eastern clade (Fig. 2B). Notably, sequences belonging to both the Western and Eastern clades were present in this same grove location at Agbofieti, Ibadan (Fig. 2B). The *argH*-specific sequences of the P-endosymbiont *Ca*. Carsonella ruddii also segregated into the previously defined Western and Eastern clades^25^ with strong (>60%) bootstrap support (Fig. 2C). Like the ACP *mtCOI* sequences, most of the Nigerian *argH* sequences (MT036086, MT036087 and MT036089) (n = 3) clustered into the Western clade, while only one sequence (MT036088 from Agbofieti, Ibadan) clustered into the Eastern clade (Fig. 2C). The results also showed that the sequenced individuals from this location belonged to both the Western and Eastern clades (Fig. 2C). Unlike the *mtCOI* and *argH* sequences, the *atpA* sequences of the S-endosymbiont *Ca*. Profftella armatura segregated into three distinct clades with strong (>60%) bootstrap support (Fig. 2D). Interestingly, most of the sequences from Nigeria (MT040184 - MT040188) (n = 5) formed a distinct clade, now referred to as ‘African’ clade, separate from the Western and Eastern clades (Fig. 2D). An additional sequence from Agbofieti, Ibadan (MT040183) clustered into the Eastern clade apart from other sequenced individuals from this same location (Fig. 2D). These results showed that whereas gene-specific sequences derived from field-collected ACP individuals and their bacterial endosymbionts in Nigeria are genetically uniform, samples from one specific location (Agbofieti, Ibadan) represented distinct populations, a likely consequence of multiple introductions of the ACP into this location. None of the ACP samples tested positive for CLaf, CLam and CLas by qPCR.

## Discussion

The results obtained in this study represent the first report of the invasive Asian citrus psyllid (*D. citri*) in Nigeria, to the best of our knowledge. Recently, the occurrence of *D. citri* was reported in East Africa from Tanzania^18^ and Kenya^19^. This has prompted ongoing intense surveillance for the pest especially in several citrus-producing eastern and southern African countries. Considering the risks associated with global movement of plant germplasm and the long history of citriculture in Nigeria, we initiated this study to determine the status of *D. citri* in Nigeria with the goal of detecting it early (if present) to inform concerted management efforts in Nigeria and across West Africa. Using a combination of morphological and molecular analysis, the occurrence of *D. citri* was confirmed from 8 spatially disparate locations in Oyo state, a major citrus-producing state in Nigeria that shares extensive land border with the Republic of Benin in West Africa.

The optimal temperature for *D. citri* development is 24–28 °C, but it has a larger temperature plasticity enabling it to survive temperature conditions ranging from 4 °C to 41 °C^26^. Using a temperature-based model of suitability, Taylor *et al*.^27^ reported that the entire continent of Africa has climate suitable for the establishment of *D. citri* and HLB. Their predictive niche map placed Nigeria and many West and Central African countries within the areas of greatest risk of HLB establishment should the disease enter these regions. Thus, the confirmation of *D. citri* in Nigeria in the present study highlights a grave threat, not only to Nigeria but also to other neighboring West African countries due to the invasive nature of ACP.

The mitochondrial *COI* (*mtCOI*) gene of *D. citri* is versatile for assessing the biodiversity within the insect’s population from different geographical regions and have been used extensively in population studies^9,28–30^. However, only few studies have assessed gene-specific sequences of *Ca*. Carsonella ruddii and *Ca*. Profftella armatura, both maternally inherited endosymbionts of *D. citri* (Wang *et al*.^25^ and references therein). Whereas the *Ca*. Carsonella ruddii like other primary endosymbionts may help provide essential amino acids that are present at low concentrations in the diets of *D. citri*^31^, *Ca*. Profftella armatura was reported as a defensive symbiont of the insect^24^. The important roles of these endosymbionts in the life of *D. citri* and the likelihood of their co-evolution with their host^32^ informed our decision to perform a comparative assessment of their encoded genes along with the host-specific gene to better decipher the evolutionary history of the sampled psyllid individuals from Nigeria. Our phylogenetic analysis revealed that the detected *D. citri* populations in Nigeria and their *Ca*. Carsonella ruddii endosymbionts mainly belonged to the Western clade that consists of psyllid populations that generally include individuals from Asia and the Americas^25,33,34^. It is therefore plausible that ACP may have been inadvertently introduced via infested plant materials from the Americas or Asia. However, unlike the congruent phylogenies of *D. citri mtCOI* and *Ca*. Carsonella ruddii *argH* (Fig. 2B,C), the *Ca*. Profftella armatura from Nigeria formed a distinct clade (Fig. 2D), indicating that they have diverged considerably from the taxa belonging to the Western and Eastern clade post-introduction. This may be an indication that *D. citri* may have been present long enough in Nigeria to allow for their local adaptation. Furthermore, the detection of a *D. citri* individual and corresponding endosymbionts belonging to the Eastern clade, a group that comprises populations restricted to Southeast Asia and South and East China, in Agbofieti, Oyo state and the occurrence of mixed variant populations in this location suggests that more than one introduction event of *D. citri* into Nigeria has occurred over time. This calls for more concerted efforts to strengthen quarantine measures to prevent further introductions of populations of this invasive pest into Nigeria and other West African countries.

Although citrus production in Nigeria has been expanding for the last few decades, there is a paucity of information on the occurrence and distribution of key citrus pests and diseases in the country. HLB has emerged as a global threat to the sustainability of citrus industries worldwide^6^ and the disease is a major concern for countries where smallholder farmers are the key players. While the HLB-associated bacteria are transmissible through grafting and vegetative propagation, psyllids play a key role in their long distance and within grove spread. Not only that *D. citri* can be moved over long distances via trade of citrus materials such as budwood, rootstock seedling or nursery plants^19^, it can naturally travel over long distances^17,35^. In many parts of the world, initial introduction and subsequent spread of the Asian form of HLB has mainly occurred through infective *D. citri* as was the case for Florida^36^, Texas^37^ and California^10^. The gravity of the possible consequence of HLB into Nigeria is underscored by the fact that the current subsistence nature of citriculture in Nigeria is antithetical to effective implementation of the recommended practices for HLB mitigation such as establishment of clean plant programs, area-wide intensive ACP management, establishment of larger contiguous blocks, etc.^6,7^. In addition, considering a recent report of CLaf from Nigeria^45^, the prospect of its mixed infections with CLas should be concerning^6^. Therefore, the findings reported here should trigger national and regional emergencies to determine the extent of *D. citri* occurrence in West Africa and to implement region-wide management efforts to control this destructive pest before it becomes endemic and prior to the incursion of CLas in the region.

## Materials and Methods

### Sample collection

Citrus pest surveys were conducted in groves and residential areas in October 2019 in Oyo state, southwestern Nigeria (Table 1) to determine the presence of *D. citri*. The survey sites were selected along major highways. A total of 10 sites (groves = 4; backyards = 6) were sampled. At each location, the citrus species was determined and a Garmin Montana 680t handheld global positioning device (Garmin, Olathe, KS, USA) was used to record the approximate sample coordinates. All the citrus trees present in backyards were inspected, while eight border trees were inspected per grove (two trees per grove perimeter). The canopy of each selected tree was visually inspected for the presence of *D. citri* life stages^20^. The young expanding flush shoots were preferentially inspected since they constitute the reproduction sites for *D. citri*^17^. The presence of feeding damage such as ‘epinasty’ or distortion of young and tender leaves due to sap uptake of *D. citri* nymphs and adults, and the presence of wax-like dropping were also used to guide inspection of flush shoots. Suspect *D. citri* adults were collected using an aspirator and nymphs were collected using a fine camel hairbrush into plastic vials containing 95% ethanol.

### Identification of *D. citri*

#### Morphological identification

Initial visual identification of the insects was made based on their morphological characteristics^20^, following which a subset of samples was sent to Texas A&M University-Kingsville Citrus Center (TAMUK-CC), Weslaco, Texas, USA for further morphological and molecular analysis. A detailed morphological characterization of the suspect insects was conducted at the Entomology Laboratory of TAMUK-CC by comparing them with archival reference voucher specimens. Morphological identification of *D. citri* nymphs and adults were carried out following features described by Mead^20^, Yang^21^ and OEPP/EPPO^22^. Voucher specimens of identified *D. citri* nymphs and adults from Nigeria were deposited at TAMUK-CC and at NIHORT, Ibadan, Nigeria.

#### Nucleic acid isolation and PCR

Total nucleic acids (TNA) was extracted from individual ACP adults and nymphs according to the Dellaporta *et al*.^38^ extraction protocol. The TNA extracts were quantified, and their quality analyzed on a NanoDrop 2000 series spectrophotometer (Thermo Fisher Scientific Inc., Waltham, MA, USA) and stored at −20 °C until use. A 2 µL aliquot of each sample was used as template in a 25 μL polymerase chain reaction (PCR) with the reagents and Rapid Protocol described for the PrimeSTAR GXLDNA Polymerase (Takara Bio USA, Inc., Mountain View, CA). The primer pair DCITRI COI-L and DCITRI COI-R were used to target an 821 bp fragment of the mtCOI coding region of each insect^29^ (Table 2). The DNA samples were also subjected to PCR using single copy housekeeping gene-specific primer pairs argH-F1/argH-R1 and atpA-F1/atpA-R1 targeting the *argH* of the psyllid primary endosymbiont (P-endosymbiont) *Ca* Carsonella ruddii and the *atpA* of the secondary endosymbiont (S-endosymbiont) *Ca* Profftella armatura, respectively (Table 2) to determine if the bacteriome of the sampled individuals from Nigeria contain these endosymbionts. DNA extracts from laboratory reared ACP individuals from TAMUK-CC were included as positive controls. The amplified products were ran on 1% agarose gels prestained with ethidium bromide along with the 100–2,000 bp Wide-Range DNA Ladder (Takara Bio USA, Inc.), followed by visualization under a UV-transilluminator.

#### Cloning and sequencing

The sample and target specific DNA bands of the correct sizes were excised and gel-eluted using the Zymoclean Gel DNA Recovery Kit (Zymo Research, Irvine, CA). The recovered DNA were ligated individually into the pJET1.2/blunt vector using the CloneJET PCR Cloning Kit (Thermo Fisher Scientific) as per the manufacturer’s recommendations. The ligation products were used to transform chemically competent DH5α *Escherichia coli* cells and two to three plasmids with PCR-verified correct size inserts per cloned DNA amplicon were isolated from recombinant *E. coli* cells using the GenElute Plasmid Miniprep Kit (Sigma-Aldrich, St. Louis, MO). Each plasmid sample was sequenced in both directions with the pJET1.2 F and pJET1.2 R primers by the Sanger method in a commercial facility (ELIM BIOPHARM, Hayward, CA, USA).

#### Bioinformatic analysis

The raw sequences were analyzed with VecScreen (https://www.ncbi.nlm.nih.gov/tools/vecscreen/) and trimmed to remove pJET1.2 contaminant. The CAP contig assembly program of the BioEdit software^39^ was used to derive a consensus sequence from each of the sample-specific forward and reverse sequences. Each of the consensus sequences was subjected to BLASTN analysis^40^ for species identification purposes. Gene-specific sequences representative of the taxon diversity were assembled from GenBank and the MUSCLE alignment program (http://www.ebi.ac.uk/Tools/msa/muscle/) was used to generate multiple sequence alignments for the sequences derived in this study and those obtained from GenBank. The gene-specific alignment files were used to determine the sequence identity matrices and for phylogenetic analysis with the maximum likelihood algorithm of the molecular evolutionary genetics analysis (MEGA) software version 7.0^41^.

### Tests for *Ca*. Liberibacter spp

The DNA extracts were assayed for CLaf, CLam and CLas using the Taqman Multiplex Real-Time PCR assays performed on ABI 7500 Fast Thermocycler (Thermo Fisher Scientific Inc., Waltham, MA) or a SmartCycler II (Cepheid, Sunnyvale, CA) and analyzed as previously described^42–44^. All reactions contained known positive and negative control DNA samples, and non-template water control. A sample was considered positive for a specific bacterium using a cycle threshold (Ct) of ≤37.

## Supplementary information

Supplementary Information.

## Data Availability

The datasets generated and/or analyzed during this study are available upon request from the corresponding author.

## References

[1] Olife IC, Ibeagha OA, Onwualu AP (2015). Citrus fruits value chain development in Nigeria. J. Biol. Agric. Healthcare.

[2] Jolaoso, M. A. et al. Citrus production and processing in Nigeria. RMRDC Monograph Series No. 003. ISBN 078-978-915-003-8 (2011).

[3] FAO. FAOSTAT. http://faostat.fao.org/ (2019).

[4] Aubert B (1987). Trioza erytreae Del Guercio and Diaphorina citri Kuwayama (Homoptera: Psylloidea), the two vectors of citrus greening disease: biological aspects and possible control strategies. Fruits.

[5] Lee RF (2015). Control of virus diseases of citrus. Adv. Virus Res..

[6] Bové JM (2006). Huanglongbing: a destructive, newly emerging, century-old disease of citrus. J. Plant Pathol..

[7] Gottwald TR (2010). Current epidemiological understanding of citrus Huanglongbing. Annu. Rev. Phytopathol..

[8] da Graça JV (1991). Citrus greening disease. Annu. Rev. Phytopathol..

[9] Grafton-Cardwell EE, Stelinski LL, Stansly PA (2013). Biology and management of Asian citrus psyllid, vector of huanglongbing pathogens. Annu. Rev. Entomol..

[10] Wang Y (2017). Genetic diversity of Diaphorina citri and its endosymbionts across east and southeast Asia. Pest Manag. Sci..

[11] Saponari M (2010). First report of Candidatus Liberibacter asiaticus associated with Huanglongbing in sweet Orange in Ethiopia. Plant Dis..

[12] Wang CL (1981). Ecological studies of Asiatic citrus psyllid (Diaphorina citri Kuwayama) with special reference to its spatial distribution. J. Agric. Res. China.

[13] French JV, Kahlke CJ, da Graça JV (2001). First record of the Asian citrus psylla Diaphorina citri Kuwayama (Homoptera:Psyllidae), in Texas. Subtrop. Plant Sci..

[14] Yamamoto PT, Paiva PEB, Gravena S (2001). Population dynamics of Diaphorina citri Kuwayama (Hemiptera: Psyllidae) in citrus orchards in the North of Sao Paulo State, Brazil. Neotrop. Entomol..

[15] Rodríguez-Palomera M, Cambero-Campos J, Robles-Bermúdez A, Carvajal-Cazola C, Estrada-Virgen O (2012). Associated natural enemies of Diaphorina citri Kuwayama (Hemiptera: Psyllidae) in Persian lime (Citrus latifolia Tanaka) in Nayarit. México. Acta Zool. Mexicana.

[16] EPPO. PQR database. Paris, France: European and Mediterranean Plant Protection Organization. http://www.eppo.int/DATABASES/pqr/pqr.htm (2014).

[17] Sétamou M, Flores D, French JV, Hall DG (2008). Dispersion patterns and sampling plans for Diaphorina citri (Hemiptera: Psyllidae) in citrus. J. Econ. Entomol..

[18] Shimwela MM (2016). First occurrence of Diaphorina citri in East Africa, characterization of the Ca. Liberibacter species causing Huanglongbing (HLB) in Tanzania, and potential further spread of D. citri and HLB in Africa and Europe. Eur. J. Plant Pathol..

[19] Rwomushana I (2017). Detection of Diaphorina citri Kuwayama (Hemiptera: Liviidae) in Kenya and potential implication for the spread of Huanglongbing disease in East Africa. Biol. Invasions.

[20] Mead, F. W. The Asiatic citrus psyllid, Diaphorina citri Kuwayama (Homoptera: Psyllidae). Florida Department of Agriculture Conservation Service, Div. Plant Ind. Entomol. Circ. 180, 1–3. http://www.freshfromflorida.com/pi/enpp/ento/entcirc/ent180.pdf (1977).

[21] Yang CT (1984). Psyllidae of Taiwan. Taiwan Mus. Spec. Publ. Ser..

[22] OEPP/EPPO (2005). EPPO Standards PM 7/52(1). Diagnostic protocol for Diaphorina citri. OEPP/EPPO Bull..

[23] Thao ML, Clark MA, Burckhardt DH, Moran NA, Baumann P (2001). Phylogenetic analysis of vertically transmitted psyllid endosymbionts (Candidatus Carsonella ruddii) based on atpAGD and rpoC: comparisons with 16S-23S rDNA-derived phylogeny. Curr. Microbiol..

[24] Nakabachi A (2013). Defensive bacteriome symbiont with a drastically reduced. Curr. Biol..

[25] Wang Y (2018). Phylogeography of Diaphorina citri (Hemiptera: Liviidae) and its primary endosymbiont, ‘Candidatus Carsonella ruddii’: an evolutionary approach to host-endosymbiont interaction. Pest Manag. Sci..

[26] Hall DG, Richardson ML, Ammar ED, Halbert SE (2013). Asian citrus psyllid, Diaphorina citri, vector of citrus Huanglongbing disease. Entomol. Exp. Appl..

[27] Taylor RA (2018). Predicting the fundamental thermal niche of crop pests and diseases in a changing world: a case study on citrus greening. J. Appl. Ecol..

[28] de León JH (2011). Two separate introductions of Asian citrus psyllid populations found in the American continents. Ann. Entomol. Soc. Am..

[29] Boykin LM (2012). Overview of worldwide diversity of Diaphorina citri Kuwayama mitochondrial cytochrome oxidase 1 haplotypes: two old world lineages and a new world invasion. Bull. Entomol. Res..

[30] Guidolin AS, Fresia P, Cônsoli FL (2014). The genetic structure of an invasive pest, the Asian citrus psyllid Diaphorina citri (Hemiptera: Liviidae). PLoS ONE.

[31] Moran NA (2007). Symbiosis as an adaptive process and source of phenotypic complexity. Proc. Natl. Acad. Sci. USA.

[32] Hall AA (2016). Codivergence of the primary bacterial endosymbiont of psyllids versus host switches and replacement of their secondary bacterial endosymbionts. Environ. Microbiol..

[33] Saha S (2012). Survey of endosymbionts in the Diaphorina citri metagenome and assembly of a Wolbachia wDi draft genome. PLoS ONE.

[34] Lashkari M (2014). Global genetic variation in the Asian citrus psyllid, Diaphorina citri (Hemiptera: Liviidae) and the endosymbiont Wolbachia: links between Iran and the USA detected. Pest Manag. Sci..

[35] Martini X, Rivera M, Hoyte A, Sétamou M, Stelinski LL (2018). Effects of wind, temperature, and barometric pressure on Asian citrus psyllid (Hemiptera: Liviidae) flight behavior. J. Econ. Entomol..

[36] Halbert, S. E. The discovery of huanglongbing in Florida. Pages 7-11 in: Proc. 2nd International Citrus Canker and Huanglongbing Res Workshop, Orlando, FL (2005).

[37] Sétamou, M., Olufemi, J. A., Kunta, M., Dale, J. & da Graça, J. V. Distribution of Candidatus Liberibacter asiaticus in citrus and the Aisan citrus psyllid in Texas over a decade. *Plant Dis*. **104**, 1118–1126, 10.1094/PDIS-08-19-1779-RE (2020). 10.1094/PDIS-08-19-1779-RE 32040392

[38] Dellaporta SL, Wood J, Hicks JB (1983). A plant DNA mini preparation: Version II. Plant Mol. Biol. Rep..

[39] Hall TA (1999). BioEdit: a user-friendly biological sequence alignment editor and analysis program for Windows 95/98/NT. Nucleic Acid Symp..

[40] Altschul SF, Gish W, Miller W, Myers EW, Lipman DJ (1990). Basic local alignment search tool. J. Mol. Biol..

[41] Kumar S, Stecher G, Tamura K (2016). MEGA7: Molecular evolutionary genetics analysis version 7.0 for bigger datasets. Mol. Biol. Evol..

[42] Li W, Hartung JS, Levy L (2006). Quantitative real-time PCR for detection and identification of Candidatus Liberibacter species associated with citrus huanglongbing. J. Microbiol. Meth..

[43] Li, W., Duan, Y., Brlansky, R., Twieg, E. & Levy, L. Incidences and population of ‘Candidatus Liberibacter asiaticus’ in Asian citrus psyllid (Diaphorina citri) on citrus plants affected by huanglongbing in Florida. *Int. Res. Conf. HLB*, Dec. 1-5, 2008, Orlando, Florida. (2008a).

[44] Li W, Li D, Twieg E, Hartung JS, Levy L (2008). Optimized quantification of unculturable Candidatus Liberibacter spp. in host plants using real-time PCR. Plant Dis..

[45] Ajene, I. J., *et al*. First report of ‘Candidatus Liberibacter africanus’ associated with citrus greening disease in Nigeria. *Plant Dis*. **104**, 1535–1535, 10.1094/PDIS-11-19-2380-PDN (2020).

