# Supplementary material for: First report of the occurrence of Asian citrus psyllid Diaphorina citri (Hemiptera: Liviidae), an invasive species in Nigeria, West Africa
Source: Sci Rep. 2020 Jun 10;10:9418. doi: 10.1038/s41598-020-66380-4 (PMC7287059; doi:10.1038/s41598-020-66380-4)
Supplement: Supplementary file 1 — Supplementary Information. [file 41598_2020_66380_MOESM1_ESM.pdf]

First report of the occurrence of Asian citrus psyllid *Diaphorina citri* (Hemiptera: Liviidae), an invasive species in Nigeria, West Africa

Abiola Olufunke Oke[1], Abiola Adeyinka Oladigbolu[1], Madhurababu Kunta[2], Olufemi J. Alabi[3], and Mamoudou Sétamou[2]*

[1]National Horticultural Research Institute (NIHORT), Idi-Ishin Jericho Reservation Area, P.M.B. 5432, Ibadan, Nigeria

[2]Texas A&M University-Kingsville Citrus Center, Weslaco 78599

[3]Department of Plant Pathology & Microbiology, Texas A&M AgriLife Research & Extension Center, Weslaco, TX 78596

*Corresponding authors:

Dr. Mamoudou Sétamou

Texas A&M University-Kingsville Citrus Center

312 N International Blvd, Weslaco, TX 78599

mamoudou.setamou@tamuk.edu

mtCOI sequences

>MT040168-ONA-6-[Nigeria]

TACATTCTTATTCTTCCGGGGTTTGGGTTAATCTCACATATTACTACACAAGAAAGAGGTAAAA
CTTCAGCATTCGGTACATTAGGAATAATTTATGCTATATTAGCTATTGGAATTTTAGGATTTAT
TGTATGAGCTCACCACATATTTACTGTAGGAATAGATGTAGACTCACGAGCTTATTTTACTTCA
GCAACCATAATTATTGCAGTACCGACAGGAATTAAAATTTTTAGTTGACTAGCTACAATTTACG
GAATAAAAATAAAATTTTCTCCAAGTATTTCATGATCATTAGGATTTATCTTCCTTTTTACAGT
AGGAGGACTTACAGGAGTAATTTTAGCAAATTCATCAATTGACATTATTCTTCATGACACTTAT
TATGTAGTTGCCCACTTTCACTATGTGCTATCAATAGGGGCTGTATTTGCCATTATTGCTAGAT
TTATTAATTGATACCCTTTATTAACAGGAAATACAATAAATAAAACTTTACTTAAAGCTCAATT
TTTAAGAACCTTCGTCGGAGTAAACACTACCTTTTTCCCCCAACACTTCTTAGGACTTATAGGA
ATGCCACGACGATACTCGAACTATCCAGATCTTCTCATTTTCTGAAATATTATTTCTTCCTTAG
GGTCTATAATTTCACTATTTTCAGTATTACTACTTATAATCATTATCTGAGAAGCAATAACTTC
TAATCGAATTGTTTTATTCAATAGA

>MT040169-ONA-9-[Nigeria]

TACATTCTTATTCTTCCGGGGTTTGGGTTAATCTCACATATTACTACACAAGAAAGAGGTAAAA
CTTCAGCATTCGGTACATTAGGAATAATTTATGCTATATTAGCTATTGGAATTTTAGGATTTAT
TGTATGAGCTCACCACATATTTACTGTAGGAATAGATGTAGACTCACGAGCTTATTTTACTTCA
GCAACCATAATTATTGCAGTACCGACAGGAATTAAAATTTTTAGTTGACTAGCTACAATTTACG
GAATAAAAATAAAATTTTCTCCAAGTATTTCATGATCATTAGGATTTATCTTCCTTTTTACAGT
AGGAGGACTTACAGGAGTAATTTTAGCAAATTCATCAATTGACATTATTCTTCATGACACTTAT
TATGTAGTTGCCCACTTTCACTATGTGCTATCAATAGGGGCTGTATTTGCCATTATTGCTAGAT
TTATTAATTGATACCCTTTATTAACAGGAAATACAATAAATAAAACTTTACTTAAAGCTCAATT
TTTAAGAACCTTCGTCGGAGTAAACACTACCTTTTTCCCCCAACACTTCTTAGGACTTATAGGA
ATGCCACGACGATACTCGAACTATCCAGATCTTCTCATTTTCTGAAATATTATTTCTTCCTTAG
GGTCTATAATTTCACTATTTTCAGTATTACTACTTATAATCATTATCTGAGAAGCAATAACTTC
TAATCGAATTGTTTTATTCAATAGA

>MT040170-ONA-12-[Nigeria]

TACATTCTTATTCTTCCGGGGTTTGGGTTAATCTCACATATTACTACACAAGAAAGAGGTAAAA
CTTCAGCATTCGGTACATTAGGAATAATTTATGCTATATTAGCTATTGGAATTTTAGGATTTAT
TGTATGAGCTCACCACATATTTACTGTAGGAATAGATGTAGACTCACGAGCTTATTTTACTTCA
GCAACCATAATTATTGCAGTACCGACAGGAATTAAAATTTTTAGTTGACTAGCTACAATTTACG
GAATAAAAATAAAATTTTCTCCAAGTATTTCATGATCATTAGGATTTATCTTCCTTTTTACAGT
AGGAGGACTTACAGGAGTAATTTTAGCAAATTCATCAATTGACATTATTCTTCATGACACTTAT
TATGTAGTTGCCCACTTTCACTATGTGCTATCAATAGGGGCTGTATTTGCCATTATTGCTAGAT
TTATTAATTGATACCCTTTATTAACAGGAAATACAATAAATAAAACTTTACTTAAAGCTCAATT
TTTAAGAACCTTCGTCGGAGTAAACACTACCTTTTTCCCCCAACACTTCTTAGGACTTATAGGA
ATGCCACGACGATACTCGAACTATCCAGATCTTCTCATTTTCTGAAATATTATTTCTTCCTTAG
GGTCTATAATTTCACTATTTTCAGTATTACTACTTATAATCATTATCTGAGAAGCAATAACTTC
TAATCGAATTGTTTTATTCAATAGA

>MT040171-ONB-8-[Nigeria]

TACATTCTTATTCTTCCGGGGTTTGGGTTAATCTCACATATTACTACACAAGAAAGAGGTAAAA
CTTCAGCATTCGGTACATTAGGAATAATTTATGCTATATTAGCTATTGGAATTTTAGGATTTAT
TGTATGAGCTCACCACATATTTACTGTAGGAATAGATGTAGACTCACGAGCTTATTTTACTTCA
GCAACCATAATTATTGCAGTACCGACAGGAATTAAAATTTTTAGTTGACTAGCTACAATTTACG
GAATAAAAATAAAATTTTCTCCAAGTATTTCATGATCATTAGGATTTATCTTCCTTTTTACAGT
AGGAGGACTTACAGGAGTAATTTTAGCAAATTCATCAATTGACATTATTCTTCATGACACTTAT
TATGTAGTTACCCACTTTCACTATGTGCTATCAATAGGGGCTGTATTTGCCATTATTGCTAGAT
TTATTAATTGATACCCTTTATTAACAGGAAATACAATAAATAAAACTTTACTTAAAGCTCAATT
TTTAAGAACCTTCGTCGGAGTAAACACTACCTTTTTCCCCCAACACTTCTTAGGACTTATAGGA
ATGCCACGACGATACTCGAACTATCCAGATCTTCTCATTTTCTGAAATATTATTTCTTCCTTAG
GGTCTATAATTTCACTATTTTCAGTATTACTACTTATAATCATTATCTGAGAAGCAATAACTTC
TAATCGAATTGTTTTATTCAATAGA

>MT040172-ONB-11-[Nigeria]

TACATTCTTATTCTTCCGGGGTTTGGGTTAATCTCACATATTACTACACAAGAAAGAGGTAAAA
CTTCAGCATTCGGTACATTAGGAATAATTTATGCTATATTAGCTATTGGAATTTTAGGATTTAT
TGTATGAGCTCACCACATATTTACTGTAGGAATAGATGTAGACTCACGAGCTTATTTTACTTCA
GCAACCATAATTATTGCAGTACCGACAGGAATTAAAATTTTTAGTTGACTAGCTACAATTTACG
GAATAAAAATAAAATTTTCTCCAAGTATTTCATGATCATTAGGATTTATCTTCCTTTTTACAGT
AGGAGGACTTACAGGAGTAATTTTAGCAAATTCATCAATTGACATTATTCTTCATGACACTTAT
TATGTAGTTGCCCACTTTCACTATGTGCTATCAATAGGGGCTGTATTTGCCATTATTGCTAGAT
TTATTAATTGATACCCTTTATTAACAGGAAATACAATAAATAAAACTTTACTTAAAGCTCAATT
TTTAAGAACCTTCGTCGGAGTAAACACTACCTTTTTCCCCCAACACTTCTTAGGACTTATAGGA
ATGCCACGACGATACTCGAACTATCCAGATCTTCTCATTTTCTGAAATATTATTTCTTCCTTAG
GGTCTATAATTTCACTATTTTCAGTATTACTACTTATAATCATTATCTGAGAAGCAATAACTTC
TAATCGAATTGTTTTATTCAATAGA

>MT040173-ONB-12-[Nigeria]

TACATTCTTATTCTTCCGGGGTTTGGGCTAATCTCACATATCACTACACAAGAAAGAGGTAAAA
CTTCAGCATTCGGTACATTAGGAATAATTTATGCTATATTAGCTATTGGGATTTTAGGATTTAT
TGTATGAGCTCACCACATATTTACTGTAGGAATAGATGTAGACTCACGAGCTTATTTTACTTCA
GCAACCATAATTATTGCAGTATCGACAGGAATTAAAATTTTTAGTTGACTAGCTACAATTTACG
GAATAAAAATAAAATTTTCTCCAAGTATTTCATGATCATTAGGATTTATCTTCCTTTTTACAGT
AGGAGGACTTACAGGAGTAATTTTAGCAAATTCATCAATTGACATTATTCTTCATGACACTTAT
TATGTAGTTGCCCACTTTCACTATGTGCTATCAATAGGGGCTGTATTTGCCATTATTGCTAGAT
TTATTAATTGATACCCTTTATTAACAGGAAATACAATAAATAAAACTTTACTTAAAGCTCAATT
TTTAAGAACCTTCGTCGGAGTAAACACTACCTTTTTCCCCCAACACTTCTTAGGACTTATAGGA
ATGCCACGACGTTACTCGAACTATCCAGATCTTCTCATTTTCTGGAATATTATTTCTTCCTTAG
GGTCTATAATTTCACTATTTTCAGTATTACTACTTATAATCATTATCTGAGAAGCAATAACTTC
TAATCGAATTGTTTTATTCAATAGA

>MT040174-ONC-2-[Nigeria]

TACATTCTTATTCTTCCGGGGTTTGGGTTAATCTCACATATTACTACACAAGAAAGAGGTAAAA
CTTCAGCATTCGGTACATTAGGAATAATTTATGCTATATTAGCTATTGGAATTTTAGGATTTAT
TGTATGAGCTCACCACATATTTACTGTAGGAATAGATGTAGACTCACGAGCTTATTTTACTTCA
GCAACCATAATTATTGCAGTACCGACAGGAATTAAAATTTTTAGTTGACTAGCTACAATTTACG
GAATAAAAATAAAATTTTCTCCAAGTATTTCATGATCATTAGGATTTATCTTCCTTTTTACAGT
AGGAGGACTTACAGGAGTAATTTTAGCAAATTCATCAATTGACATTATTCTTCATGACACTTAT
TATGTAGTTGCCCACTTTCACTATGTGCTATCAATAGGGGCTGTATTTGCCATTATTGCTAGAT
TTATTAATTGATACCCTTTATTAACAGGAAATACAATAAATAAAACTTTACTTAAAGCTCAATT
TTTAAGAACCTTCGTCGGAGTAAACACTACCTTTTTCCCCCAACACTTCTTAGGACTTATAGGA
ATGCCACGACGATACTCGAACTATCCAGATCTTCTCATTTTCTGAAATATTATTTCTTCCTTAG
GGTCTATAATTTCACTATTTTCAGTATTACTACTTATAATCATTATCTGAGAAGCAATAACTTC
TAATCGAATTGTTTTATTCAATAGA

>MT040175-ONC-5-[Nigeria]

TACATTCTTATTCTTCCGGGGTTTGGGTTAATCTCACATATTACTACACAAGAAAGAGGTAAAA
CTTCAGCATTCGGTACATTAGGAATAATTTATGCTATATTAGCTATTGGAATTTTAGGATTTAT
TGTATGAGCTCACCACATATTTACTGTAGGAATAGATGTAGACTCACGAGCTTATTTTACTTCA
GCAACCATAATTATTGCAGTACCGACAGGAATTAAAATTTTTAGTTGACTAGCTACAATTTACG
GAATAAAAATAAAATTTTCTCCAAGTATTTCATGATCATTAGGATTTATCTTCCTTTTTACAGT
AGGAGGACTTACAGGAGTAATTTTAGCAAATTCATCAATTGACATTATTCTTCATGACACTTAT
TATGTAGTTGCCCACTTTCACTATGTGCTATCAATAGGGGCTGTATTTGCCATTATTGCTAGAT
TTATTAATTGATACCCTTTATTAACAGGAAATACAATAAATAAAACTTTACTTAAAGCTCAATT
TTTAAGAACCTTCGTCGGAGTAAACACTACCTTTTTCCCCCAACACTTCTTAGGACTTATAGGA
ATGCCACGACGATACTCGAACTATCCAGATCTTCTCATTTTCTGAAATATTATTTCTTCCTTAG
GGTCTATAATTTCACTATTTTCAGTATTACTACTTATAATCATTATCTGAGAAGCAATAACTTC
TAATCGAATTGTTTTATTCAATAGA

>MT040176-ONC-11-[Nigeria]

TACATTCTTATTCTTCCGGGGTTTGGGTTAATCTCACATATTACTACACAAGAAAGAGGTAAAA
CTTCAGCATTCGGTACATTAGGAATAATTTATGCTATATTAGCTATTGGAATTTTAGGATTTAT
TGTATGAGCTCACCACATATTTACTGTAGGAATAGATGTAGACTCACGAGCTTATTTTACTTCA
GCAACCATAATTATTGCAGTACCGACAGGAATTAAAATTTTTAGTTGACTAGCTACAATTTACG
GAATAAAAATAAAATTTTCTCCAAGTATTTCATGATCATTAGGATTTATCTTCCTTTTTACAGT
AGGAGGACTTACAGGAGTAATTTTAGCAAATTCATCAATTGACATTATTCTTCATGACACTTAT
TATGTAGTTGCCCACTTTCACTATGTGCTATCAATAGGGGCTGTATTTGCCATTATTGCTAGAT
TTATTAATTGATACCCTTTATTAACAGGAAATACAATAAATAAAACTTTACTTAAAGCTCAATT
TTTAAGAACCTTCGTCGGAGTAAACACTACCTTTTTCCCCCAACACTTCTTAGGACTTATAGGA
ATGCCACGACGATACTCGAACTATCCAGATCTTCTCATTTTCTGAAATATTATTTCTTCCTTAG
GGTCTATAATTTCACTATTTTCAGTATTACTACTTATAATCATTATCTGAGAAGCAATAACTTC
TAATCGAATTGTTTTATTCAATAGA

>MT040177-ONCn-34-[Nigeria]

TACATTCTTATTCTTCCGGGGTTTGGGTTAATCTCACATATTACTACACAAGAAAGAGGTAAAA
CTTCAGCATTCGGTACATTAGGAATAATTTATGCTATATTAGCTATTGGAATTTTAGGATTTAT
TGTATGAGCTCACCACATATTTACTGTAGGAATAGATGTAGACTCACGAGCTTATTTTACTTCA
GCAACCATAATTATTGCAGTACCGACAGGAATTAAAATTTTTAGTTGACTAGCTACAATTTACG
GAATAAAAATAAAATTTTCTCCAAGTATTTCATGATCATTAGGATTTATCTTCCTTTTTACAGT
AGGAGGACTTACAGGAGTAATTTTAGCAAATTCATCAATTGACATTATTCTTCATGACACTTAT
TATGTAGTTGCCCACTTTCACTATGTGCTATCAATAGGGGCTGTATTTGCCATTATTGCTAGAT
TTATTAATTGATACCCTTTATTAACAGGAAATACAATAAATAAAACTTTACTTAAAGCTCAATT
TTTAAGAACCTTCGTCGGAGTAAACACTACCTTTTTCCCCCAACACTTCTTAGGACTTATAGGA
ATGCCACGACGATACTCGAACTATCCAGATCTTCTCATTTTCTGAAATATTATTTCTTCCTTAG
GGTCTATAATTTCACTATTTTCAGTATTACTACTTATAATCATTATCTGAGAAGCAATAACTTC
TAATCGAATTGTTTTATTCAATAGA

>MT040178-ONCn-35-[Nigeria]

TACATTCTTATTCTTCCGGGGTTTGGGTTAATCTCACATATTACTACACAAGAAAGAGGTAAAA
CTTCAGCATTCGGTACATTAGGAATAATTTATGCTATATTAGCTATTGGAATTTTAGGATTTAT
TGTATGAGCTCACCACATATTTACTGTAGGAATAGATGTAGACTCACGAGCTTATTTTACTTCA
GCAACCATAATTATTGCAGTACCGACAGGAATTAAAATTTTTAGTTGACTAGCTACAATTTACG
GAATAAAAATAAAATTTTCTCCAAGTATTTCATGATCATTAGGATTTATCTTCCTTTTTACAGT
AGGAGGACTTACAGGAGTAATTTTAGCAAATTCATCAATTGACATTATTCTTCATGACACTTAT
TATGTAGTTGCCCACTTTCACTATGTGCTATCAATAGGGGCTGTATTTGCCATTATTGCTAGAT
TTATTAATTGATACCCTTTATTAACAGGAAATACAATAAATAAAACTTTACTTAAAGCTCAATT
TTTAAGAACCTTCGTCGGAGTAAACACTACCTTTTTCCCCCAACACTTCTTAGGACTTATAGGA
ATGCCACGACGATACTCGAACTATCCAGATCTTCTCATTTTCTGAAATATTATTTCTTCCTTAG
GGTCTATAATTTCACTATTTTCAGTATTACTACTTATAATCATTATCTGAGAAGCAATAACTTC
TAATCGAATTGTTTTATTCAATAGA

>MT040179-ONCn-36-[Nigeria]

TACATTCTTATTCTTCCGGGGTTTGGGTTAATCTCACATATTACTACACAAGAAAGAGGTAAAA
CTTCAGCATTCGGTACATTAGGAATAATTTATGCTATATTAGCTATTGGAATTTTAGGATTTAT
TGTATGAGCTCACCACATATTTACTGTAGGAATAGATGTAGACTCACGAGCTTATTTTACTTCA
GCAACCATAATTATTGCAGTACCGACAGGAATTAAAATTTTTAGTTGACTAGCTACAATTTACG
GAATAAAAATAAAATTTTCTCCAAGTATTTCATGATCATTAGGATTTATCTTCCTTTTTACAGT
AGGAGGACTTACAGGAGTAATTTTAGCAAATTCATCAATTGACATTATTCTTCATGACACTTAT
TATGTAGTTGCCCACTTTCACTATGTGCTATCAATAGGGGCTGTATTTGCCATTATTGCTAGAT
TTATTAATTGATACCCTTTATTAACAGGAAATACAATAAATAAAACTTTACTTAAAGCTCAATT
TTTAAGAACCTTCGTCGGAGTAAACACTACCTTTTTCCCCCAACACTTCTTAGGACTTATAGGA
ATGCCACGACGATACTCGAACTATCCAGATCTTCTCATTTTCTGAAATATTATTTCTTCCTTAG
GGTCTATAATTTCACTATTTTCAGTATTACTACTTATAATCATTATCTGAGAAGCAATAACTTC
TAATCGAATTGTTTTATTCAATAGA

>MT040180-ONCn-37-[Nigeria]

TACATTCTTATTCTTCCGGGGTTTGGGTTAATCTCACATATTACTACACAAGAAAGAGGTAAAA
CTTCAGCATTCGGTACATTAGGAATAATTTATGCTATATTAGCTATTGGAATTTTAGGATTTAT
TGTATGAGCTCACCACATATTTACTGTAGGAATAGATGTAGACTCACGAGCTTATTTTACTTCA
GCAACCATAATTATTGCAGTACCGACAGGAATTAAAATTTTTAGTTGACTAGCTACAATTTACG
GAATAAAAATAAAATTTTCTCCAAGTATTTCATGATCATTAGGATTTATCTTCCTTTTTACAGT
AGGAGGACTTACAGGAGTAATTTTAGCAAATTCATCAATTGACATTATTCTTCATGACACTTAT
TATGTAGTTGCCCACTTTCACTATGTGCTATCAATAGGGGCTGTATTTGCCATTATTGCTAGAT
TTATTAATTGATACCCTTTATTAACAGGAAATACAATAAATAAAACTTTACTTAAAGCTCAATT
TTTAAGAACCTTCGTCGGAGTAAACACTACCTTTTTCCCCCAACACTTCTTAGGACTTATAGGA
ATGCCACGACGATACTCGAACTATCCAGATCTTCTCATTTTCTGAAATATTATTTCTTCCTTAG
GGTCTATAATTTCACTATTTTCAGTATTACTACTTATAATCATTATCTGAGAAGCAATAACTTC
TAATCGAATTGTTTTATTCAATAGA

>MT040181-TX-1-[USA:Texas]

TACATTCTTATTCTTCCGGGGTTTGGGTTAATCTCACATATTACTACACAAGAAAGAGGTAAAA
CTTCAGCATTCGGTACATTAGGAATAATTTATGCTATATTAGCTATTGGAATTTTAGGATTTAT
TGTATGAGCTCACCACATATTTACTGTAGGAATAGATGTAGACTCACGAGCTTATTTTACTTCA
GCAACCATAATTATTGCAGTACCGACAGGAATTAAAATTTTTAGTTGACTAGCTACAATTTACG
GAATAAAAATAAAATTTTCTCCAAGTATTTCATGATCATTAGGATTTATCTTCCTTTTTACAGT
AGGAGGACTTACAGGAGTAATTTTAGCAAATTCATCAATTGACATTATTCTTCATGACACTTAT
TATGTAGTTGCCCACTTTCACTATGTGCTATCAATAGGGGCTGTATTTGCCATTATTGCTAGAT
TTATTAATTGATACCCTTTATTAACAGGAAATACAATAAATAAAACTTTACTTAAAGCTCAATT
TTTAAGAACCTTCGTCGGAGTAAACACTACCTTTTTCCCCCAACACTTCTTAGGACTTATAGGA
ATGCCACGACGATACTCGAACTATCCAGATCTTCTCATTTTCTGAAATATTATTTCTTCCTTAG
GGTCTATAATTTCACTATTTTCAGTATTACTACTTATAATCATTATCTGAGAAGCAATAACTTC
TAATCGAATTGTTTTATTCAATAGA

>MT040181-TX-1-[USA:Texas]

TACATTCTTATTCTTCCGGGGTTTGGGTTAATCTCACATATTACTACACAAGAAAGAGGTAAAA
CTTCAGCATTCGGTACATTAGGAATAATTTATGCTATATTAGCTATTGGAATTTTAGGATTTAT
TGTATGAGCTCACCACATATTTACTGTAGGAATAGATGTAGACTCACGAGCTTATTTTACTTCA
GCAACCATAATTATTGCAGTACCGACAGGAATTAAAATTTTTAGTTGACTAGCTACAATTTACG
GAATAAAAATAAAATTTTCTCCAAGTATTTCATGATCATTAGGATTTATCTTCCTTTTTACAGT
AGGAGGACTTACAGGAGTAATTTTAGCAAATTCATCAATTGACATTATTCTTCATGACACTTAT
TATGTAGTTGCCCACTTTCACTATGTGCTATCAATAGGGGCTGTATTTGCCATTATTGCTAGAT
TTATTAATTGATACCCTTTATTAACAGGAAATACAATAAATAAAACTTTACTTAAAGCTCAATT
TTTAAGAACCTTCGTCGGAGTAAACACTACCTTTTTCCCCCAACACTTCTTAGGACTTATAGGA
ATGCCACGACGATACTCGAACTATCCAGATCTTCTCATTTTCTGAAATATTATTTCTTCCTTAG
GGTCTATAATTTCACTATTTTCAGTATTACTACTTATAATCATTATCTGAGAAGCAATAACTTC
TAATCGAATTGTTTTATTCAATAGA

argH sequences

>MT036086-ONA-12-[Nigeria]

TCTTGGAATGAAATGTTAAAAAGAGACCATTTAAATTTACTAAATTGCAAAAAAAGTTTACAAT
ATTGCCCTTTAGGATCTGCAGCTTTATCAGGACATAATTACAATATTAATAGAAATACTATTAA
AAAATTTTTAAATTTTAAAAATTTAACAGAAAATTCTGTAGATGCAGTAAGCGATAGAGATTAT
ATTGTTATGTTTGCTCATTTTTGTAATTTAATTATTACTCATTTATCTAGAATATCTGAAGATA
TGATAATTTGGAGTAATAATAACTTTGATTTTTTAAAATTATCAGATTTGATTTCTTCTGGTTC
TTCAATAATGCCTCAAAAAAAAAATCCTGATTTATTTGAATTAATAAGAGCAAAAACAGGAAGA
ATATATGGAAATTCTTTAAGTATTTTAACTATTTTAAAAGCTCAACCGTTGTCATATAATAAAG
ATAATCAAGAAGACAAAGAAAGTCTCTTCGATAATGTTTATACAATTAAAAAAACTCTTAATTC
TTTTAGAAAATGCTTACCAATTTTAAAATTTAATAAAAAAAACATGTATTTTTCGGCTTTGAAA
AATTATTCAACAGCAACAGACATGGCTGATTATTTAGTAAAAAAAGGTGTTTTATTTCGAGAAG
CACACAAAATAGTAGGTAATTGTATTCAATATTGTGAAAAAAATAATATTAATTTGTTTAATAT
TTCTTTAAACGAATTAAAAAGATTTAGTAATTTATTTGAAAAAAATATTTTTTATGATTTATC

>MT036087-ONA-14-[Nigeria]

TCTTGGAATGAAATGTTAAAAAGAGACCATTTAAATTTACTAAATTGCAAAAAAAGTTTACAAT
ATTGCCCTTTAGGATCTGCAGCTTTATCAGGACATAATTACAATATTAATAGAAATACTATTAA
AAAATTTTTAAATTTTAAAAATTTAACAGAAAATTCTGTAGATGCAGTAAGCGATAGAGATTAT
ATTGTTATGTTTGCTCATTTTTGTAATTTAATTATTACTCATTTATCTAGAATATCTGAAGATA
TGATAATTTGGAGTAATAATAACTTTGATTTTTTAAAATTATCAGATTTGATTTCTTCTGGTTC
TTCAATAATGCCTCAAAAAAAAAATCCTGATTTATTTGAATTAATAAGAGCAAAAACAGGAAGA
ATATATGGAAATTCTTTAAGTATTTTAACTATTTTAAAAGCTCAACCGTTGTCATATAATAAAG
ATAATCAAGAAGACAAAGAAAGTCTCTTCGATAATGTTTATACAATTAAAAAAACTCTTAATTC
TTTTAGAAAATGCTTACCAATTTTAAAATTTAATAAAAAAAACATGTATTTTTCGGCTTTGAAA
AATTATTCAACAGCAACAGACATGGCTGATTATTTAGTAAAAAAAGGTGTTTTATTTCGAGAAG
CACACAAAATAGTAGGTAATTGTATTCAATATTGTGAAAAAAATAATATTAATTTGTTTAATAT
TTCTTTAAACGAATTAAAAAGATTTAGTAATTTATTTGAAAAAAATATTTTTTATGATTTATC

>MT036088-ONB-20-[Nigeria]

TCTTGGAATGAAATGTTAAAAAGAGATCATTTAAATTTACTAAATTGCAAAAAAAGTTTACAAT
ATTGCCCTTTAGGATCTGCAGCTTTATCAGGACATAATTACAATATTAATAGAAATACTATTAA
AAAATTTTTAAATTTTAAAAATTTAACAGAAAATTCTGTAGATGCAGTAAGCGATAGAGATTAT
ATTGTTATGTTTGCTCATTTTTGTAATTTAATTATTACTCATTTATCTAGAATATCTGAAGATA
TGATAATTTGGAGTAATAATAACTTTGATTTTTTAAAATTATCAGATTTGATTTCTTCTGGTTC
TTCAATAATGCCTCAAAAAAAAAATCCTGATTTATTTGAATTAATAAGAGCAAAAACAGGAAGA
ATATATGGAAATTCTTTAAGTATTTTAACTATTTTAAAAGCTCAACCGTTGTCATATAATAAAG
ATAATCAAGAAGACAAAGAAAGTCTCTTCGATAATGTTTATACAATTAAAAAAACTCTTAATTC
TTTTAGAAAATGCTTACCAATTTTAAAATTTAATAAAAAAAACATGTATTTTTCGGCTTTGAAA
AATTATTCAACAGCAACAGACATGGCTGATTATTTAGTAAAAAAAGGTGTTTTATTTCGAGAAG
CACACAAAATAGTAGGTAATTGTATTCAATATTGTGAAAAAAATAATATTAATTTGTTTAATAT
TTCTTTAAACGAATTAAAAAGATTTAGTAATTTATTTGAAAAAAATATTTTTTATGATTTATC

>MT036089-ONB-21-[Nigeria]

TCTTGGAATGAAATGTTAAAAAGAGACCATTTAAATTTACTAAATTGCAAAAAAAGTTTACAAT
ATTGCCCTTTAGGATCTGCAGCTTTATCAGGACATAATTACAATATTAATAGAAATACTATTAA
AAAATTTTTAAATTTTAAAAATTTAACAGAAAATTCTGTAGATGCAGTAAGCGATAGAGATTAT
ATTGTTATGTTTGCTCATTTTTGTAATTTAATTATTACTCATTTATCTAGAATATCTGAAGATA
TGATAATTTGGAGTAATAATAACTTTGATTTTTTAAAATTATCAGATTTGATTTCTTCTGGTTC
TTCAATAATGCCTCAAAAAAAAAATCCTGATTTATTTGAATTAATAAGAGCAAAAACAGGAAGA
ATATATGGAAATTCTTTAAGTATTTTAACTATTTTAAAAGCTCAACCGTTGTCATATAATAAAG
ATAATCAAGAAGACAAAGAAAGTCTCTTCGATAATGTTTATACAATTAAAAAAACTCTTAATTC
TTTTAGAAAATGCTTACCAATTTTAAAATTTAATAAAAAAAACATGTATTTTTCGGCTTTGAAA
AATTATTCAACAGCAACAGACATGGCTGATTATTTAGTAAAAAAAGGTGTTTTATTTCGAGAAG
CACACAAAATAGTAGGTAATTGTATTCAATATTGTGAAAAAAATAATATTAATTTGTTTAATAT
TTCTTTAAACGAATTAAAAAGATTTAGTAATTTATTTGAAAAAAATATTTTTTATGATTTATC

atpA sequences

>MT040183-ONB-16-[Nigeria]

GATATAGTTAAATGTACAGGACGTATTTTAGAAGTTCCTATTGGCCCGGAATTATGTGGTCGAG
TCATTAATGCTCTTGGTGATCCAATTGACGGAAAAGGGCCAATTAAAACAAAATTAACTGCTCC
AATTGAAAAAGTTGCTCCCGGAGTAATTTCTCGACAATCAGTCTCCGAACCCCTGCAAACAGGT
ATTAAAGCAATAGATTCTATAGTGCCAATTGGAAAAGGTCAACGTGAATTAATTATTGGAGATA
GACAAACTGGAAAAATCTTCCATAGCAATAGACATAATTATTAATCAAAAAAATAAAAATGTTAC
TTGTATTTATGTTGCTATTGGACAAAAGATATCATCAATTAAAAAAACCGCAAATTTATTAGAA
AAATATGGAGCAATGCCATATACTATTATTGTAGCTGCAACAGCTTCTGATTCTGCTTCTATGC
AATTTATATCAGCTTATTCAGGTTGTACTATTGGAGAATATTTTAGAGATCATGGAAAAGATGC
ATTAGTTGTTTATGACGATTTATCTAAACAAGCTGTTGCTTATCGTCAAATATCATTACTCTTA
AAACGACCACCAGGAAGAGAGGCTTATCCTGGAGATATATTTTATTTACATAGTCGATTATTAG
AACGCTCAGCTAGAGTAAATATTAAATATGTAGAAAGTTATACAAACGGTAAAGTAACTGGTAA
AACTGGATCTTTAAC

>MT040184-ONB-18-[Nigeria]

GATATAGTTAAATGTACAGGACGTATTTTAGAAGTTCCTATTGGCCCGGAATTATGTGGTCGAG
TCATTAATGCTCTTGGTGATCCAATTGACGGAAAAGGGCCAATTAAAACAAAATTAACTGCTCC
AATTGAAAAAGTTGCTCCCGGAGTAATTTCTCGACAATCAGTCTCCGAACCCCTGCAAACAGGT
ATTAAAGCAATAGATTCTATAGTGCCAATTGGAAAAGGTCAACGTGAATTAATTATTGGAGATA
GACAAACTGGAAAAATCTTCCATAGCAATAGACATAATTATTAATCAAAAAAATAAAAATGTTAC
TTGTATTTATGTTGCTATTGGACAAAAGATATCATCAATTAAAAAAACCGCAAATTTATTAGAA
AAATATGGAGCAATGCCATATACTATTATTGTAGCTGCAACAGCTTCTGATTCTGCTTCTATGC
AATTTATATCAGCTTATTCAGGTTGTACTATTGGAGAATATTTTAGAGATCATGGAAAAGATGC
ATTAGTTGTTTATGACGATTTATCTAAACAAGCTGTTGCTTATCGTCAAATATCATTACTCTTA
AAACGACCACCAGGAAGAGAGGCTTATCCTGGGGATATATTTTATTTACATAGTCGATTATTAG
AACGCTCAGCTAGAGTAAATATTAAATATGTAGAAAATTATACAAACGGTAAAGTAACTGGTAA
AACTGGATCTTTAAC

>MT040185-ONC-25-[Nigeria]

GATATAGTTAAATGTACAGGACGTATTTTAGAAGTTCCTATTGGCCCGGAATTATGTGGTCGAG
TCATTAATGCTCTTGGTGATCCAATTGACGGAAAAGGGCCAATTAAAACAAAATTAACTGCTCC
AATTGAAAAAGTTGCTCCCGGAGTAATTTCTCGACAATCAGTCTCCGAACCCCTGCAAACAGGT
ATTAAAGCAATAGATTCTATAGTGCCAATTGGAAAAGGTCAACGTGAATTAATTATTGGAGATA
GACAAACTGGAAAATCTTCCATAGCAATAGACATAATTATTAATCAAAAAAATAAAAATGTTAC
TTGTATTTATGTTGCTATTGGACAAAAGATATCATCAATTAAAAAAACCGCAAATTTATTAGAA
AAATATGGAGCAATGCCATATACTATTATTGTAGCTGCAACAGCTTCTGATTCTGCTTCTATGC
AATTTATATCAGCTTATTCAGGTTGTACTATTGGAGAATATTTTAGAGATCATGGAAAAGATGC
ATTAGTTGTTTATGACGATTTATCTAAACAAGCTGTTGCTTATCGTCAAATATCATTACTCTTA
AAACGACCACCAGGAAGAGAGGCTTATCCTGGGGATATATTTTATTTACATAGTCGATTATTAG
AACGCTCAGCTAGAGTAAATATTAAATATGTAGAAAATTATACAAACGGTAAAGTAACTGGTAA
AACTGGATCTTTAAC

>MT040186-ONC-28-[Nigeria]

GATATAGTTAAATGTACAGGACGTATTTTAGAAGTTCCTATTGGCCCGGAATTATGTGGTCGAG
TCATTAATGCTCTTGGTGATCCAATTGACGGAAAAGGGCCAATTAAAACAAAATTAACTGCTCC
AATTGAAAAAGTTGCTCCCGGAGTAATTTCTCGACAATCAGTCTCCGAACCCCTGCAAACAGGT
ATTAAAGCAATAGATTCTATAGTGCCAATTGGAAAAGGTCAACGTGAATTAATTATTGGAGATA
GACAAACTGGAAAATCTTCCATAGCAATAGACATAATTATTAATCAAAAAAATAAAAATGTTAC
TTGTATTTATGTTGCTATTGGACAAAAGATATCATCAATTAAAAAAACCGCAAATTTATTAGAA
AAATATGGAGCAATGCCATATACTATTATTGTAGCTGCAACAGCTTCTGATTCTGCTTCTATGC
AATTTATATCAGCTTATTCAGGTTGTACTATTGGAGAATATTTTAGAGATCATGGAAAAGATGC
ATTAGTTGTTTATGACGATTTATCTAAACAAGCTGTTGCTTATCGTCAAATATCATTACTCTTA
AAACGACCACCAGGAAGAGAGGCTTATCCTGGGGATATATTTTATTTACATAGTCGATTATTAG
AACGCTCAGCTAGAGTAAATATTAAATATGTAGAAAATTATACAAACGGTAAAGTAACTGGTAA
AACTGGATCTTTAAC

>MT040187-ONCn-30H-[Nigeria]

GATATAGTTAAATGTACAGGACGTATTTTAGAAGTTCCTATTGGCCCGGAATTATGTGGTCGAG
TCATTAATGCTCTTGGTGATCCAATTGACGGAAAAGGGCCAATTAAAACAAAATTAACTGCTCC
AATTGAAAAAGTTGCTCCCGGAGTAATTTCTCGACAATCAGTCTCCGAACCCCTGCAAACAGGT
ATTAAAGCAATAGATTCTATAGTGCCAATTGGAAAAGGTCAACGTGAATTAATTATTGGAGATA
GACAAACTGGAAAATCTTCCATAGCAATAGACATAATTATTAATCAAAAAAATAAAAATGTTAC
TTGTATTTATGTTGCTATTGGACAAAAGATATCATCAATTAAAAAAACCGCAAATTTATTAGAA
AAATATGGAGCAATGCCATATACTATTATTGTAGCTGCAACAGCTTCTGATTCTGCTTCTATGC
AATTTATATCAGCTTATTCAGGTTGTACTATTGGAGAATATTTTAGAGATCATGGAAAAGATGC
ATTAGTTGTTTATGACGATTTATCTAAACAAGCTGTTGCTTATCGTCAAATATCATTACTCTTA
AAACGACCACCAGGAAGAGAGGCTTATCCTGGGGATATATTTTATTTACATAGTCGATTATTAG
AACGCTCAGCTAGAGTAAATATTAAATATGTAGAAAATTATACAAACGGTAAAGTAACTGGTAA
AACTGGATCTTTAAC

>MT040188-ONCn-31H-[Nigeria]

GATATAGTTAAATGTACAGGACGTATTTTAGAAGTTCCTATTGGCCCGGAATTATGTGGTCGAG
TCATTAATGCTCTTGGTGATCCAATTGACGGAAAAGGGCCAATTAAAACAAAATTAACTGCTCC
AATTGAAAAAGTTGCTCCCGGAGTAATTTCTCGACAATCAGTCTCCGAATCCCTGCAAACAGGT
ATTAAAGCAATAGATTCTATAGTGCCAATTGGAAAAGGTCAACGTGAATTAATTATTGGAGATA
GACAAACTGGAAAATCTTCCATAGCAATAGACATAATTATTAATCAAAAAAATAAAAATGTTAC
TTGTATTTATGTTGCTATTGGACAAAAGATATCATCAATTAAAAAAACCGCAAATTTATTAGAA
AAATATGGAGCAATGCCATATACTATTATTGTAGCTGCAACAGCTTCTGATTCTGCTTCTATGC
AATTTATATCAGCTTATTCAGGTTGTACTATTGGAGAATATTTTAGAGATCATGGAAAAGATGC
ATTAGTTGTTTATGACGATTTATCTAAACAAGCTGTTGCTTATCGTCAAATATCATTACTCTTA
AAACGACCACCAGGAAGAGAGGCTTATCCTGGGGATATATTTTATTTACATAGTCGATTATTAG
AACGCTCAGCTAGAGTAAATATTAAATATGTAGAAAATTATACAAACGGTAAAGTAACTGGTAA
AACTGGATCTTTAAC

>MT040189-TX-7-[USA:Texas]

GATATAGTTAAATGTACAGGACGTATTTTAGAAGTTCCTATTGGCCCGGAATTATGTGGTCGAG
TCATTAATGCTCTTGGTGATCCAATTGACGGAAAAGGGCCAATTAAAACAAAATTAACTGCTCC
AATTGAAAAAGTTGCTCCCGGAGTAATTTCTCGACAATCAGTCTCCGAACCCCTGCAAACAGGT
ATTAAAGCAATAGATTCTATAGTGCCAATTGGAAAAGGTCAACGTGAATTAATTATTGGAGATA
GACAAACTGGAAAATCTTCCATAGCAATAGACATAATTATTAATCAAAAAAATAAAAATGTTAC
TTGTATTTATGTTGCTATTGGACAAAAGATATCATCAATTAAAAAAACCGCAAATTTATTAGAA
AAATATGGAGCAATGCCATATACTATTATTGTAGCTGCAACAGCTTCTGATTCTGCTTCTATGC
AATTTATATCAGCTTATTCAGGTTGTACTATTGGAGAATATTTTAGAGATCATGGAAAAGATGC
ATTAGTTGTTTATGACGATTTATCTAAACAAGCTGTTGCTTATCGTCAAATATCATTACTCTTA
AAACGACCACCAGGAAGAGAGGCTTATCCTGGAGATATATTTTATTTACATAGTCGATTATTAG
AACGCTCAGCTAGAGTAAATATTAAATATGTAGAAAATTATACAAACGGTAAAGTAACTGGTAA
AACTGGATCTTTAAC

>MT040190-TX-8-[USA:Texas]

GATATAGTTAAATGTACAGGACGTATTTTAGAAGTTCCTATTGGCCCGGAATTATGTGGTCGAG
TCATTAATGCTCTTGGTGATCCAATTGACGGAAAAGGGCCAATTAAAACAAAATTAACTGCTCC
AATTGAAAAAGTTGCTCCCGGAGTAATTTCTCGACAATCAGTCTCCGAACCCCTGCAAACAGGT
ATTAAAGCAATAGATTCTATAGTGCCAATTGGAAAAGGTCAACGTGAATTAATTATTGGAGATA
GACAAACTGGAAAATCTTCCATAGCAATAGACATAATTATTAATCAAAAAAATAAAAATGTTAC
TTGTATTTATGTTGCTATTGGACAAAAGATATCATCAATTAAAAAAACCGCAAATTTATTAGAA
AAATATGGAGCAATGCCATATACTATTATTGTAGCTGCAACAGCTTCTGATTCTGCTTCTATGC
AATTTATATCAGCTTATTCAGGTTGTACTATTGGAGAATATTTTAGAGATCATGGAAAAGATGC
ATTAGTTGTTTATGACGATTTATCTAAACAAGCTGTTGCTTATCGTCAAATATCATTACTCTTA
AAACGACCACCAGGAAGAGAGGCTTATCCTGGAGATATATTTTATTTACATAGTCGATTATTAG
AACGCTCAGCTAGAGTAAATATTAAATATGTAGAAAATTATACAAACGGTAAAGTAACTGGTAA
AACTGGATCTTTAAC
